# Assessing frailty at the centers for dementia and cognitive decline in Italy: potential implications for improving care of older people living with dementia

**DOI:** 10.1007/s10072-023-06885-8

**Published:** 2023-06-06

**Authors:** G. Bellelli, A. Zucchelli, A. Benussi, E. Pinardi, S. Caratozzolo, A.M. Ornago, M. Cosseddu, V. Stella, R. Turrone, F. Massariello, A. Marengoni, A. Padovani

**Affiliations:** 1 School of Medicine and Surgery, University of Milano-Bicocca, Milan, Italy; 2 Fondazione IRCCS San Gerardo dei Tintori, Via Pergolesi 33, 20900 Monza, Italy; 3 Department of Clinical and Experimental Sciences, Geriatric Unit, University of Brescia, Brescia, Italy; 4 Department of Clinical and Experimental Sciences, Neurology Clinic, University of Brescia, Brescia, Italy

**Keywords:** Cognitive disorders, Dementia, Elderly, Frailty, Frailty index, Personalized medicine

## Abstract

**Introduction:**

Frailty is strongly associated with the clinical course of cognitive impairment and dementia, thus arguing for the need of its assessment in individuals affected by cognitive deficits. This study aimed to retrospectively evaluate frailty in patients aged 65 years and older referred to two Centers for Cognitive Decline and Dementia (CCDDs).

**Methods:**

A total of 1256 patients consecutively referred for a first visit to two CCDDs in Lombardy (Italy) between January 2021 to July 2022 were included. All patients were evaluated by an expert physician in diagnosis and care of dementia according to a standardized clinical protocol. Frailty was assessed using a 24-items Frailty Index (FI) based on routinely collected health records, excluding cognitive decline or dementia, and categorized as mild, moderate, and severe.

**Results:**

Overall, 40% of patients were affected by mild frailty and 25% of the sample has moderate to severe frailty. The prevalence and severity of frailty increased with decreasing Mini Mental State Examination (MMSE) score and advancing age. Frailty was also detected in 60% of patients with mild cognitive impairment.

**Conclusion:**

Frailty is common in patients referring to CCDDs for cognitive deficits. Its systematic assessment using a FI generated with readily available medical information could help develop appropriate models of assistance and guide personalization of care.

**Supplementary Information:**

The online version contains supplementary material available at 10.1007/s10072-023-06885-8.

## Introduction

There are over 500 Centers for Cognitive Decline and Dementia (CCDDs) in Italy, with 72 of them located in the Lombardy Region. These centers, established in 2001 and previously known as Alzheimer’s Evaluation Units, are integrated into the National and Regional Health Systems and are primarily led by neurologists and geriatricians. Most patients are referred to CCDDs by general practitioners to undergo timely differential diagnosis and receive appropriate treatment of dementia and related behavioral disturbances [1]. CCDDs are responsible for prescribing anti-dementia drugs and other pharmacological treatments required for managing behavioral disturbances of dementia, as well as for providing advice and support to caregivers. Patients referred to CCDDs undergo standardized clinical assessments for the diagnosis and care of cognitive disturbances. However, the evaluation of frailty is not routinely performed.

Frailty is a geriatric syndrome characterized by the decline of multiple organs and systems, leading to an individual having increased vulnerability to adverse clinical events, such as falls, hospitalization, loss of function, and death [2]. Advanced age does not necessarily equate to frailty. However, its prevalence sharply increases in the oldest population [3]. Frailty is frequently operationalized using the deficit accumulation approach, which postulates that an individual’s frailty level is related to the extent of the health deficits that he/she has accumulated during the life course, and is expressed as a single continuous variable called the Frailty Index (FI) [4]. There is growing evidence that frailty is more prevalent in cognitively impaired patients [5] and associated with the development of cognitive disorders in unimpaired individuals. Using data from the English Longitudinal Study of Ageing, Rogers and colleagues demonstrated that a 47-item FI predicted cognitive decline and incident dementia among cognitively intact older individuals [6]. Similarly, in a study of 7239 cognitively intact community-dwelling older adults, a 19-item FI predicted the incidence of Alzheimer’s disease (AD) and dementia over 10 years [7]. Importantly, frailty not only appears to influence the relationship between neuropathology and clinical presentation of dementia in AD but is even more informative for dementia in individuals with low AD pathology [8, 9].

Therefore, incorporating the evaluation of frailty in the assessment of patients attending CCDDs may help provide a more comprehensive view of patients’ global health status and unmet needs, improve the reliability of predicting dementia development and its clinical course, and allow for the appropriate allocation of healthcare resources.

The aim of this study was to assess the presence of frailty in patients aged 65 years and older who were referred for their first visit to two CCDDs (Lombardy, Italy), one led by neurologists and the other by geriatricians.

## Methods

### Setting and population

This is a retrospective study of patients who attended their initial visit at the CCDDs in Brescia and Monza (Lombardy region, Italy) between January 1st, 2021 and July 31st, 2022.. Both CCDDs are hospital-based and regularly serve a median of 180 individuals per month. The staff is comprised of neurologists at the Brescia CCDD, geriatricians at the Monza CCDD, and neuropsychologists at both centers. These CCDDs have access to several advanced diagnostic procedures, including brain computed tomography (CT) and magnetic resonance imaging (RMN), electroencephalography, fluorodeoxyglucose positron emission tomography (FDG-PET), and cerebrospinal fluid (CSF) biomarkers.

Patients were included in the study provided they were aged 65 years or older and attending the CCDDs for the first visit. Exclusion criteria were attending a visit for reasons other than cognitive deficits and related disorders, seeking civil invalidity and disability certification, or being unable to speak Italian.

The following information was collected for all included participants: demographic data (age, gender, and years of education), information on cognitive (Mini Mental State Examination, MMSE [10]) and functional (activities of daily living, ADL [11]; Instrumental Activities of Daily Living, IADL [12]) status, a list of chronic diseases, and a list of medications used. MMSE score was further adjusted for age and educational level using the score-adjustment coefficients derived by the previous analysis on a large Italian older population [13]. Frailty was assessed using a Frailty Index which was recently developed and validated by Vetrano and colleagues, using information that is readily available in the software employed by general practitioners in Italy [14]. For the aims of this research, presence/absence of the FI items was determined retrospectively by extracting from the electronic databases routinely used by the two CCDDs to record patients’ clinical information. Given the lack of information about patients’ financial difficulties, 24 of the 25 original variables were assessed (Online Resource 1). To simulate an evaluation based on the information available to a general practitioner, all patients were assumed to be free from cognitive decline or dementia, which accounted for one of the potential deficits included in the score. The FI total score was computed by calculating the ratio between the number of deficits that were observed in the individual and the total number of considered items. The score ranged from 0 (no deficit) to 1 (all deficits are present).

This study was conducted as part of standard care activities and was performed according to Good Clinical Practices guidelines.

### Statistical analysis

Descriptive analyses were conducted to present the characteristics of the study sample. Participants were categorized into four groups on the basis of their frailty level, according to cut-off points previously defined in the work by Vetrano et al [14]. In detail, FI < 0.07 defined patients as non-frail, 0.07 ≤ FI < 0.14 as mildly frail, 0.14 ≤ FI < 0.21 as moderately frail and FI ≥ 21 as severely frail. Participants with missing information about 2 or more deficits were excluded (*n* = 19). Data are presented as mean (SD) and median (IQR) for continuous variables or percentage (%) for categorical variables. Unpaired two-sided heteroscedastic *t* tests and two-sided chi-square tests were performed to compare the results. The analyses reported in Online Resource 2 are based on a linear mixed model including CCDD as random effect and all independent variables as fixed effect. The contrasts matrix was personalized in order to evaluate the change in mean MMSE from each frailty category in comparison with the previous one (e.g., mild frailty vs no frailty, moderate frailty vs mild frailty, and so on). All analyses were performed using R version 4.2.1.

## Results

Overall, 1256 patients were included in the analyses, 815 from CCDDs in Brescia and 441 from CCDD in Monza. The mean age was 78.5 (SD 6.2) years, 40.8% were males, and the mean years of education was 7.5 (SD 3.7). A substantial proportion of patients were dependent in the activities of daily living (44%) and in the instrumental activities of daily living (69%). As shown in Table 1, 35.4% of patients exhibited no frailty, 40% had mild frailty and nearly 25% showed moderate-to-severe frailty. The two CCDDs differed for frailty severity, as well as for age, sex, education level, and disability burden. Patients evaluated in Monza also demonstrated a lower mean MMSE raw score; however, the observed difference became statistically non-significant after score adjustment by age and education.

**Table 1:** Demographic and clinical characteristics of the whole sample and by CCDDs

	All	CDCD; BS	CDCD; MB	*p*
*n*	1256	815	441	
Age, years, mean (SD)	78.5 (6.2)	76.8 (5.9)	81.8 (5.4)	< 0.001
Sex, males, *n* (%)	512 (40.8)	352 (43.2)	160 (36.3)	0.020
Education, years, mean (SD)	7.5 (3.7)	7.8 (3.8)	6.8 (3.4)	< 0.001
ADL, 0 function lost, *n* (%)	687 (54.7)	502 (61.6)	185 (42.0)	< 0.001
1	304 (24.2)	181 (22.2)	123 (27.9)	
2–4	210 (16.7)	98 (12.0)	112 (25.4)	
5–6	55 (4.4)	34 (4.2)	21 (4.8)	
IADL, 1+ functions lost, *n* (%)	865 (68.9)	538 (66.0)	327 (74.1)	0.004
MMSE score, mean (SD)	22.4 (5.5)	22.6 (5.3)	22.0 (5.8)	0.044
MMSE adj. score, mean (SD)	22.7 (5.2)	22.7 (5.0)	22.8 (5.4)	0.742
FI, median (Q1–Q3)	0.1 (0.0-0.1)	0.1 (0.0-0.1)	0.1 (0.0-0.2)	< 0.001
No frailty, *n* (%)	445 (35.4)	320 (39.3)	125 (28.3)	< 0.001
Mild frailty, *n* (%)	197 (15.7)	124 (15.2)	73 (16.6)	
Severe frailty, *n* (%)	112 (8.9)	58 (7.1)	54 (12.2)	
Cerebrovascular diseases, *n* (%)	112 (8.9)	73 (9.0)	39 (8.8)	1.000
Malignancy, *n* (%)	238 (18.9)	147 (18.0)	91 (20.6)	0.296
COPD, *n* (%)	82 (6.5)	44 (5.4)	38 (8.6)	0.037
Coronary heart disease, *n* (%)	194 (15.4)	128 (15.7)	66 (15.0)	0.791
Chronic kidney disease, *n* (%)	203 (16.2)	151 (18.5)	52 (11.8)	0.003
Hip fracture, *n* (%)	70 (5.6)	55 (6.7)	15 (3.4)	0.019
Anemia, *n* (%)*	119 (11.4)	69 (8.5)	50 (22.2)	< 0.001
Type 2 diabetes, *n* (%)	265 (21.1)	185 (22.7)	80 (18.1)	0.069
Atrial fibrillation, *n* (%)	160 (12.7)	90 (11.0)	70 (15.9)	0.018

*AD* = activities of daily living, *IADL* = Instrumental Activities of Daily Living, *MMSE* = Mini-Mental State Examination; *FI* = Frailty Index, COPD = chronic obstructive pulmonary disease, *Q1* = 1st quartile; *Q3* = 3rd quartile; *CCDD BS* = Center for Dementia and Cognitive Decline in Brescia, Italy; *CCDD MB* = Center for Dementia and Cognitive Decline in Monza, Italy

*Missing 216 observations

According to cognitive status, the prevalence of severe frailty varied between 7.2% among those with a MMSE score of 24 or higher to 24.2% among patients with MMSE score lower than 10. In contrast, the proportion of those without frailty ranged between 39.6% among patients scoring at least 24 at the MMSE and 18.2% among those with a score lower than 10.

**Fig. 1:**
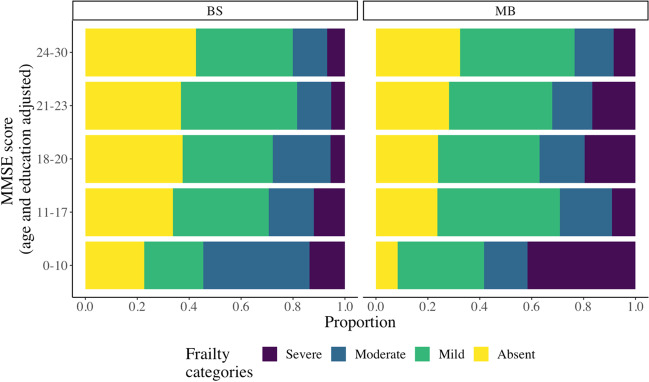
Barplot showing the proportion of different frailty categories according to MMSE categories. BS = Brescia, MB = Monza

Figure 1 shows the proportion of patients affected by frailty of various degrees in the two CCDDs according to MMSE score, after adjusting for age and education level. The percentage of participants living with frailty increased as the MMSE score declined in both cohorts, with severe frailty reaching the highest percentage in individuals with MMSE score < 10 (41.7% in Monza, 14.3% in Brescia). Notably, within all other MMSE strata, the prevalence pattern of frailty levels was similar between the two centers. Each frailty category (no frailty, mild, moderate, and severe frailty) was associated with a reduction in MMSE score even after adjusting for age, sex, and education (see Online Resource 2).

## Discussion

This study found that frailty was relatively common among patients attending the two CCDDs, with only slightly more than a third having no frailty. Mild frailty was prevalent in 40%, while moderate-severe frailty affected about one fourth of the study population. Remarkably, some patients exhibited mild frailty despite severe cognitive decline, while others had severe frailty despite fairly preserved cognitive function.

A recent meta-analysis showed that the prevalence of frailty worldwide varies between 11% and 26% in community-dwelling individuals aged 50 years and above, depending on the definition used [15]. Similarly, previous research conducted across 22 European countries reported an overall estimated frailty prevalence of 18% (95% CI 15–21%) [16]. Vetrano et al. found that, among primary care patients aged 60 years and older in Italy, 51.1% of the study population was not frail, 34.2% demonstrated mild frailty, 10.9% showed moderate frailty, and 3.8% had severe frailty [14]. Our study provides new evidence showing that frailty is a significant burden in CCDDs, compared to other clinical settings or the general population.

Since referral patterns to CCDDs have been shown to vary, with centers led by neurologists typically attracting younger individuals [17], one could infer that patients attending a neurological CCDD might be less frail compared to those attending a geriatric CCDD solely based on their chronological age. However, this was not supported by the findings of this study. Surprisingly, there were only minor differences in frailty prevalence and FI distribution across most items between the two patient populations. Although frailty is commonly associated with advanced age, purely age-based criteria can be unreliable in capturing such a complex and multifactorial phenomenon. Indeed, there is vast literature showing that frailty can also occur in younger individuals, while many older adults can maintain good health and remain robust [18, 19]. Frailty has been proven to be a pivotal tool to identify those individuals whose needs of care are more complex and who would likely benefit from a comprehensive geriatric assessment and a tailored care pathway [20, 21]. Therefore, shifting towards team-based care and collaboration between neurologists and geriatricians may result in better outcomes, as patients’ needs are often complex and overlapping.

It is also worth noting that not all individuals with moderate or severe cognitive impairment had moderate to severe frailty, suggesting that targeted interventions to prevent frailty may be warranted. Studies have shown that frailty can hasten the progression of cognitive decline in people with cognitive impairment [8, 22]. Importantly, a systematic review and meta-analysis of over 14,000 participants revealed that those with both cognitive impairment and physical frailty had over a five-fold higher risk of developing dementia than those free from both conditions [23]. Once again, neurological and geriatric co-management may be advantageous in addressing specific issues of care. Geriatricians can assist with deprescribing certain medications, managing behavioral and psychological symptoms or delirium, and implementing disability prevention programs. Neurologists can provide a detailed neurological assessment, including extrapyramidal and vegetative examinations, which can help identify atypical forms of dementing illnesses and guide diagnostic work-up and therapeutic management.

The anticipated increase in the number of older persons who are expected to develop dementia in the coming years poses a significant challenge for the National Health Service, due to the substantial care requirements and associated costs of this condition [24]. In this perspective, assessing frailty in patients attending CCDDs can help guide the care pathways and support efficient resource allocation, while avoiding unnecessary interventions. For instance, individuals with mild cognitive impairment but moderate-severe frailty may be offered a syndromic diagnostic process instead of an etiological one, given that severe frailty is a strong predictor of poor survival [25]. In such cases, discussions with caregivers about patient’s living wills and end of life decisions may be more appropriate than detailed neuropsychological assessment or advanced diagnostic tools (such as FDG PET, Amyloid PET or even CSF biomarkers). This is particularly relevant in the Lombardy region, which is currently undergoing a computerization effort to digitalize all data and documents, adopt an information model based on real-time data availability and create a network of hospital and territorial services. Alongside this, a new regional diagnostic and therapeutic care pathway (DTCP) is being developed, which includes a FI as a tool for resource allocation. This approach may provide several advantages over the other presently acknowledged methodologies for evaluating frailty. First, it is possible to retrospectively build the index by taking advantage of databases created for other purposes. Second, it enables the assessment of severity, which is not possible with the phenotype model of frailty and other tools.

The main limitations of this study include its retrospective design and the involvement of a small number of CCDDs. In addition, the differences in prevalence of certain deficits between the two CCDDs may be attributed not only to dissimilarities in patient populations but also to disparities in the accessibility and precision of the data. For example, the retrospective assessment of some of the deficits used to compute the frailty index (such as anemia, edemas, ulcers, constipation, and malnutrition) might have depended on the accuracy of physician visits.

However, the main strength of the study lies in the use of a newly developed and accurate FI, specifically tailored for the Italian population. Additionally, this is the first study to systematically assess frailty in the setting of Italian CCDDs.

In conclusion, this study demonstrates that frailty is common among individuals attending CCDDs, thus arguing for making its assessment a mandatory requirement for a multidimensional approach. Generating a FI from routinely collected data offers a convenient method for measuring frailty, independent of the professional staff in charge of the CCDDS. Therefore, frailty assessment is essential to provide meaningful information and enhance the clinical decision-making process for patients affected by cognitive decline and dementia.

## Supplementary information

ESM 1: Online Resource 1: Deficits included in the FI and their prevalence in the study, according to CCDD (DOCX 18 kb)

ESM 2: Online Resource 2: Results from a linear mixed model evaluating the relationship between frailty categories and MMSE score. CCDD was considered as random effect (DOCX 16 kb)
