# Supplementary material for: Assessing frailty at the centers for dementia and cognitive decline in Italy: potential implications for improving care of older people living with dementia
Source: Neurol Sci. 2023 Jun 6;44(10):3509–14. doi: 10.1007/s10072-023-06885-8 (PMC10495473; doi:10.1007/s10072-023-06885-8)
Supplement: Supplementary file 1 — Online Resource 1: Deficits included in the FI and their prevalence in the study, according to CCDD (DOCX 18 kb) [file 10072_2023_6885_MOESM1_ESM.docx]

**Assessing frailty at the Centers for Dementia and Cognitive Decline in Italy: potential implications for improving care of older people living with dementia**

G. Bellelli MD^1,2^, A. Zucchelli MD^3^, A. Benussi MD^4^, E. Pinardi MD^1^, S. Caratozzolo MD^4^, A.M. Ornago MD^1^, M. Cosseddu MSc^4^, V. Stella MD^1^, R. Turrone MSc^4^, F. Massariello MD^2^, A. Marengoni MD PhD^3^ * and A. Padovani MD PhD^4^ *

* Co-senior authors

**Affiliations**

1. School of Medicine and Surgery, University of Milano-Bicocca, Milan, Italy

2. Fondazione IRCCS San Gerardo dei Tintori, Monza, Italy

3. Department of Clinical and Experimental Sciences, Geriatric Unit, University of Brescia, Brescia, Italy

4. Department of Clinical and Experimental Sciences, Neurology Clinic, University of Brescia, Brescia, Italy

**Journal:** Neurological Sciences

**Corresponding Author:** Giuseppe Bellelli**,** [giuseppe.bellelli@unimib.it](mailto:giuseppe.bellelli@unimib.it)

**Online Resource 1:**

Deficits included in the FI and their prevalence in the study, according to CCDD

| **Deficit** | **Assessment** | **CCDD Brescia** | **CCDD Monza** |
| --- | --- | --- | --- |
| Cognitive Decline or Dementia | Considered absent for all participants | 0 (0.0) | 0 (0.0) |
| Severe disability | 1+ ADL missing, evaluated by physician | 313 (38.4) | 256 (58.0) |
| Cerebrovascular disease | Medical history evaluated by physician | 73 (9.0) | 39 (8.8) |
| Malignancies | Medical history evaluated by physician | 147 (18.0) | 91 (20.6) |
| Chronic Obstuctive Pulmonary Disease | Medical history evaluated by physician | 44 (5.4) | 38 (8.6) |
| Ischaemic Heart Disease | Medical history evaluated by physician | 128 (15.7) | 66 (15.0) |
| Heart Failure | Medical history evaluated by physician | 16 (2.0) | 17 (3.9) |
| Chronic Kidney Disease | Medical history evaluated by physician | 151 (18.5) | 52 (11.8) |
| Atrial Fibrillation | Medical history evaluated by physician | 90 (11.0) | 70 (15.9) |
| Parkinson disease or parkinsonism | Medical history evaluated by physician | 41 (5.0) | 5 (1.1) |
| Hip fracture | Medical history evaluated by physician | 55 (6.7) | 15 (3.4) |
| Anemia | Medical history evaluated by physician | 69 (8.5) | 50 (22.2) |
| Need for long-term oxygen therapy | Medical history evaluated by physician | 1 (0.1) | 5 (1.1) |
| Recent Hospitalization | Medical history evaluated by physician | 87 (10.7) | 37 (8.4) |
| Skin ulcers | Medical history evaluated by physician | 0 (0.0) | 2 (0.5) |
| Bradycardia and other heart conduction problems | Medical history evaluated by physician | 45 (5.5) | 48 (10.9) |
| Other neurological disease | Medical history evaluated by physician | 126 (15.5) | 40 (9.1) |
| Stipsis | Medical history evaluated by physician | 180 (22.1) | 26 (5.9) |
| Recent prescription of Low Molecular Weight Heparin | Medical history evaluated by physician | 2 (0.2) | 1 (0.2) |
| Peripheral vascular disease | Medical history evaluated by physician | 74 (9.1) | 137 (31.1) |
| Malnutrition and related disease | Medical history evaluated by physician | 28 (3.4) | 87 (19.7) |
| Type 2 diabetes mellitus | Medical history evaluated by physician | 185 (22.7) | 80 (18.1) |
| Schizophrenia | Medical history evaluated by physician | 16 (2.0) | 0 (0.0) |
| Oedemas | Physical examination performed by physician | 8 (1.0) | 41 (9.3) |
