# Supplementary material for: Assessing frailty at the centers for dementia and cognitive decline in Italy: potential implications for improving care of older people living with dementia
Source: Neurol Sci. 2023 Jun 6;44(10):3509–14. doi: 10.1007/s10072-023-06885-8 (PMC10495473; doi:10.1007/s10072-023-06885-8)
Supplement: Supplementary file 2 — Online Resource 2: Results from a linear mixed model evaluating the relationship between frailty categories and MMSE score. CCDD was considered as random effect (DOCX 16 kb) [file 10072_2023_6885_MOESM2_ESM.docx]

**Assessing frailty at the Centers for Dementia and Cognitive Decline in Italy: potential implications for improving care of older people living with dementia**

G. Bellelli MD^1,2^, A. Zucchelli MD^3^, A. Benussi MD^4^, E. Pinardi MD^1^, S. Caratozzolo MD^4^, A.M. Ornago MD^1^, M. Cosseddu MSc^4^, V. Stella MD^1^, R. Turrone MSc^4^, F. Massariello MD^2^, A. Marengoni MD PhD^3^ * and A. Padovani MD PhD^4^ *

* Co-senior authors

**Affiliations**

1. School of Medicine and Surgery, University of Milano-Bicocca, Milan, Italy

2. Fondazione IRCCS San Gerardo dei Tintori, Monza, Italy

3. Department of Clinical and Experimental Sciences, Geriatric Unit, University of Brescia, Brescia, Italy

4. Department of Clinical and Experimental Sciences, Neurology Clinic, University of Brescia, Brescia, Italy

**Journal:** Neurological Sciences

**Corresponding Author:** Giuseppe Bellelli**,** [giuseppe.bellelli@unimib.it](mailto:giuseppe.bellelli@unimib.it)

**Online Resource 2:**

Results from a linear mixed model evaluating the relationship between frailty categories and MMSE score. CCDD was considered as random effect

|  | **Beta (95%CI)** |
| --- | --- |
| Mild frailty (in comparison with no frailty) | -1.64 (-2.60 - -0.66) |
| Moderate frailty (in comparison with mild frailty) | -2.86 (-4.22 - -1.49) |
| Severe frailty (in comparison with moderate frailty) | -1.63 (-3.13 - -0.10) |
| Age, yr | -0.25 (-0.29 - -0.19) |
| Education, yr | 0.32 (0.24 – 0.40) |
| Male sex | 0.50 (-0.09 – 1.09) |
